# Dynamic Myofibrillar Remodeling in Live Cardiomyocytes under Static Stretch

**DOI:** 10.1038/srep20674

**Published:** 2016-02-10

**Authors:** Huaxiao Yang, Lucas P. Schmidt, Zhonghai Wang, Xiaoqi Yang, Yonghong Shao, Thomas K. Borg, Roger Markwald, Raymond Runyan, Bruce Z. Gao

**Affiliations:** 1 Department of Bioengineering, Clemson University, Clemson, SC, USA; 2 Key Laboratory of Optoelectronic Devices and Systems of Ministry of Education and Guangdong Province, College of Optoelectronic Engineering, Shenzhen University, Shenzhen, China; 3 Department of Regenerative Medicine and Cell Biology, Medical University of South Carolina, Charleston, SC, USA; 4 Department of Cellular and Molecular Medicine, University of Arizona, Tucson, AZ, USA

## Abstract

An increase in mechanical load in the heart causes cardiac hypertrophy, either physiologically (heart development, exercise and pregnancy) or pathologically (high blood pressure and heart-valve regurgitation). Understanding cardiac hypertrophy is critical to comprehending the mechanisms of heart development and treatment of heart disease. However, the major molecular event that occurs during physiological or pathological hypertrophy is the dynamic process of sarcomeric addition, and it has not been observed. In this study, a custom-built second harmonic generation (SHG) confocal microscope was used to study dynamic sarcomeric addition in single neonatal CMs in a 3D culture system under acute, uniaxial, static, sustained stretch. Here we report, for the first time, live-cell observations of various modes of dynamic sarcomeric addition (and how these real-time images compare to static images from hypertrophic hearts reported in the literature): 1) Insertion in the mid-region or addition at the end of a myofibril; 2) Sequential addition with an existing myofibril as a template; and 3) Longitudinal splitting of an existing myofibril. The 3D cell culture system developed on a deformable substrate affixed to a stretcher and the SHG live-cell imaging technique are unique tools for real-time analysis of cultured models of hypertrophy.

In response to an increase in mechanical load (e.g., pressure and volume overload), cardiac muscle cells (cardiomyocytes) hypertrophy^1^,^2^,^3^. This increase in mechanical load may be either a normal or an abnormal process^4^. Normal processes, such as those that occur during heart development^5^ or in the adult heart during exercise^6^ and pregnancy^7^, produce physiological hypertrophy that can strengthen the heart. Abnormal processes, such as those seen in high blood pressure or heart-valve regurgitation, produce pathological hypertrophy, which may lead to heart failure^8^,^9^,^10^. Understanding the details of cardiac hypertrophy is critical to understanding heart physiology and treatment of diseases of the heart.

Myofibrillar remodeling is involved in both physiological and pathological hypertrophy^11^,^12^,^13^: In each case, the overall size of each cardiomyocyte (CM) increases through two mechanisms: The number of myofibrils increase, and the length of each myofibril increases^14^,^15^,^16^. Myofibrillar remodeling, whether it occurs during normal embryonic cardiomyocyte growth or as a pathological hypertrophic process in adulthood, is achieved structurally through sequential addition of sarcomeres, the basic unit of myofibrils^17^,^18^: Addition to the lateral margins of myofibrils increases the total number of myofibrils; addition to the ends of existing myofibrils extends their length. It has been hypothesized that the addition of sarcomeres is the primary cause of myocyte hypertrophy^16^,^17^,^19^,^20^.

Studies using samples from hypertrophic hearts revealed that increasing blood pressure (“pressure-overload hypertrophy”) caused an increase in myofibrillar number (CM thickening)^21^, whereas increasing blood volume (“volume-overload hypertrophy”) caused an increase in myofibrillar length (CM lengthening)^22^. To further explore mechanisms of hypertrophic stimuli, CMs were cultured under conditions that evoke or simulate hypertrophy *in vivo* (e.g., exertion of mechanical forces or addition of chemical agents, including growth factors, cytokines and thyroid hormones, etc.)^23^,^24^,^25^. To mimic mechanical loading, cells were cultured on an elastic membrane that was stretched^26^,^27^,^28^. To define the direction of the mechanical load, CMs were aligned in a single direction by microfabrication techniques. For example, extracellular matrix proteins were micropatterned into lines for neonatal CM attachment and alignment. It was found that hypertrophy indicators, such as atrial natriuretic factor (ANF) and Cx43 expression, increased after either transverse (lateral) or longitudinal stretch^29^. To explore sarcomeric addition under stretch, Brenda Russell’s group induced volume-overload hypertrophy of individual CMs using 10% static stretch on a textured surface with parallel grooves. After fixing the samples and staining sarcomeric components, they captured the intermediate stage of sarcomeric insertion into an existing myofibril, but not its initiation and final outcome^30^. In an attempt to observe the dynamic processes of sarcomeric addition, GFP-tagged sarcomeric proteins were traced using live-cell imaging^31^,^32^. However, tagging of sarcomeric components by this method did not prove to be a highly efficient or a uniform way of labeling myofibrillar components; in fact, it showed the potential to impair sarcomeric assembly during live-cell imaging^32^,^33^.

Thus to date, direct observation of dynamic sarcomeric addition during hypertrophy has not been reported. Accordingly, current knowledge of sarcomeric addition during hypertrophic remodeling is based mainly on static images obtained from *in vivo* hypertrophic hearts or cells cultured under hypertrophic conditions. In the absence of time-lapse images that show the dynamic process of sarcomeric addition, the mechanisms that regulate myofibrillar assembly during hypertrophy are likely to remain poorly understood^12^,^13^.

To address this issue, we developed a second-harmonic generation (SHG) confocal microscope that utilizes the intrinsic imaging signal generated by the coiled rod structure of myosin^34^ to dynamically monitor the process of sarcomeric addition in a live neonatal CM culture system without having to tag with extrinsic markers^35^,^36^. In an SHG confocal microscope, the SHG imaging signal is typically produced simultaneously with the two-photon excitation process. This generation can be viewed as a two-photon scattering process in contrast to the one photon scattering used in conventional microscopy to obtain a phase image. Because a pulsed infrared stimulation is used, less photodamage is achieved in SHG imaging. The unique feature of SHG imaging is that the signal is from polar molecules organized in noncentrosymmetric structures not from fluorophores. Consequently, it can be used to image a specific molecule that has the required molecular structure, such as a sarcomeric myosin filament, without cell staining and thus, to achieve live cell imaging. Using our custom-built two photon (2P)/SHG confocal microscope^37^, we have been studying dynamic sarcomeric addition during myofibrillar remodeling in single, living neonatal CMs under acute, uniaxial, static, sustained stretch. Here we report, for the first time, live-cell observations of various modes of dynamic sarcomeric addition and how these real-time images compare to the static images from hypertrophic hearts reported in the literature. Using this imaging technology in combination with our *in vitro*, hypertrophic cell-culture model, we have been able to observe and dynamically capture multiple, *in vivo* modes of sarcomeric addition: Sarcomeres are added to the ends and/or inserted at a site in the mid-region of a myofibril, thus elongating it. Similarly, the parallel addition of new sarcomeres that were aligned with the Z-lines and/or to sites along the longitudinal axis where splitting of existing myofibrils occurred, causes a lateral expansion of the resident myofibrils.

## Materials and Methods

### Cell preparation

Sprague-Dawley (SD) neonatal rats (Day 3) were euthanized according to a procedure approved by Clemson University Institutional Animal Care and Use Committee (protocol number AUP2013-035). The procedure conforms to the Guide for the Care and Use of Laboratory Animals (NIH Publication, 8th Edition, 2011). The methods of euthanasia for neonatal animals are consistent with the recommendations of the Panel on Euthanasia of the American Veterinary Medical Association. Neonatal ventricular CMs were harvested from the rats using a two-day protocol. The detailed procedure is described in our previous publications^38^,^39^.

### A 3D culture model

**Figure 1:**
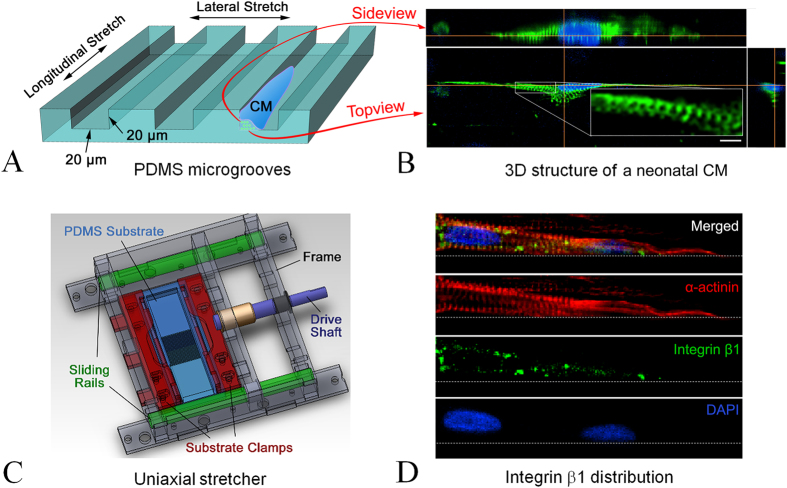
3D stretch culture model. (**A**) Dimensions of PDMS microgrooves; arrows indicate the direction of uniaxial loads. (**B**) 3D confocal image of a neonatal CM inside the microgroove: green, α-actinin; blue, cell nucleus; the insert shows a zoomed-in image of the aligned sarcomeric structure; scale bar: 10 μm. (**C**) Structure of the uniaxial cell stretcher. (**D**) Confocal image of integrin distribution on a neonatal CM inside a microgroove (a 2D image showing the distribution on the bottom surface, side wall distribution not shown).

The model was achieved by seeding individual neonatal cardiomyocytes into microgrooves as shown in the cartoon (Fig. 1A, not to scale) and developed in 3 steps: 1) Microgroove fabrication was accomplished using soft lithography to generate an elastomeric poly(dimethylsiloxane) (PDMS) substrate. In brief, a computer-aided design (AutoCAD) application was used to create the features of the microgrooves. The features were printed onto a film (mask) and transferred to a 20 μm-thick mold made of a SU-8 2025 negative photoresist on a silicon wafer. Transfer of the microgroove’s features from the mask to the mold was achieved through UV exposure using a Karl-Suss MJB aligner with a 200 W lamp house. The exposed SU-8 photoresist was then washed with SU-8 developer and dried on a hot plate to form the master mold with a relief structure of the microgrooves. Next, a highly biocompatible PDMS was prepared by mixing the base agent and the curing agent 9:1. The mixture was poured onto the SU-8 mold and cured on a hot plate at 85 °C for 2 h, and a 100 μm thick “biochip” was then carved out of the cured PDMS to produce a cell-culture substrate, on the surface of which the microgrooves were cast from the SU 8 mold. Microgroove dimensions were 20 (width) × 2000 (length) × 20 (depth) μm (Fig. 1A).

2) Collagen gel coating: Collagen type I gel was used to coat the surface of the microgrooves. The detailed method is described in our previous publication^40^.

3) Cell culture: 1 ml neonatal ventricular CMs (7.5 × 10^5^/ml) in high-glucose Dulbecco’s modified Eagle’s medium with 10% fetal bovine serum were cultured in the microgrooves for 5 days. Medium was refreshed every other day.

### Design of the uniaxial cell stretcher

The uniaxial cell stretcher (Fig. 1C) has a main frame with sliding rails and a drive shaft connected to the moving part to control the stretch of the PDMS substrate. The ratio of the lateral to the longitudinal substrate is approximately 4:1 to ensure that the cell culture that was arranged in the center of the membrane would experience solely a 1D stretch. A longitudinal mode is achieved when the microgrooves are fabricated along the axis of stretch; a lateral mode is achieved when the microgrooves are perpendicular to the axis of stretch, (Fig. 1A). At the beginning of each experiment, the degree of stretch was set to approximately 6% by turning the drive shaft to a calibrated position. The calibration was conducted by using the phase microscopy channel of our imaging system to visualize the displacement of the edges of the grooves that served as deformation markers. All the experiments were conducted at 6% acute, static, sustained stretch.

### Live cell imaging

Cells were cultured for 5 days in the microgrooves on the PDMS (biochip) membrane that was affixed to the cell stretcher and housed in a standard cell incubator (5% CO_2_ and 37 °C) and then, the entire device including the cell culture was placed in an on-stage incubator mounted on the imaging stage of the customized 2 P/SHG confocal microscope. The setup of the 2 P/SHG microscope was described previously^37^. The 2 P channel was utilized to image mitochondrial distribution (for these experiments, neonatal ventricular CMs were fluorescently labeled with MitoTracker Red (Life Technologies, US)), and the SHG channel was used to image sarcomeric patterns. During live-cell imaging, cells were live-imaged at each time point at 10 min intervals immediately after acute stretch (0 min). For each 2D image, two more focal planes were scanned (one 2 μm above the image plane and one 2 μm below). Images of these two planes were stored for future analysis to ensure that the sarcomeric addition phenomena recorded were not caused by out-of-focus effects. To obtain a clear view of the striated structure of the myofibril while the cell was contracting, a gated technique was used. The fast scan axis, along the myofibril alignment, formed a row of images, and the slow scan axis, perpendicular to the alignment, formed a column of images. In contrast to a conventional raster scan in which each row of the 2D image is scanned (slow scan) consecutively from top to bottom, in our system, each row was scanned (fast scan) repeatedly for many periods of cell contraction to reconstruct a diastolic myofibrillar structure. After every line was scanned in such a manner, an entire 2D image was reconstructed. The total imaging experiment lasted up to 4 h at each region of interest (ROI).

### Immunocytostaining

After 5 days in culture, cells in the microgrooves on the biochip attached to the stretcher were fixed with 4% paraformaldehyde (pH 7.4) solution for 15 minutes and blocked with blocking solution (10% (v/v) donkey serum and 0.25% Triton X-100 in PBS solution) for 30 min at room temperature (RT). To confirm the role of integrin in mechanical signal transduction at the cellular level, the 3D cell cultures were labeled with an integrin antibody. Samples were incubated with 1 ml mouse-anti-rat integrin β1 PBS solution (1:200, Abcam, US) at 4 °C overnight and further incubated with 1 ml Alexa Fluor^®^ 488 conjugated IgG donkey-anti-mouse PBS solution (1:200, Life Technologies, US) at RT for 1.5 h in a dark room. Next, cells were incubated with 1 ml mouse-anti-rat α-actinin PBS solution (1:500, Sigma, US) overnight and then incubated with 1 ml Alexa Fluor^®^ 546 conjugated IgG donkey-anti-mouse PBS solution (1:200, Life Technologies, US) at RT for 1.5 h in a dark room. Finally, the sample was covered with ProLong^©^ Antifade Kit mounting medium with DAPI (Life Technologies, US) for fluorescence imaging later. Cells growing inside the microgrooves on the stretcher without stretch (negative control group) were treated identically.

### Confocal imaging

A Nikon Eclipse Ti confocal microscope was used for 3D imaging of α-actinin and integrin β1 structure on the single neonatal CMs inside the microgrooves. A multichannel system was used to image integrin β1 (green), α-actinin (red), and nuclei (blue) simultaneously. In this paper, only images in Fig. 1 were obtained using this Nikon confocal microscope; other images were from our custom-built SHG confocal microscope.

### Image processing

Plugin Stackreg provided with ImageJ (HIN, USA) was used for sequential image alignment. The images from the 2p (MitoTracker Red labeled mitochondria) were binarized using the Triangle method, and then the region of interest and the entire cell on the same focal plane were analyzed in the same way using Measure in ImageJ software. Sarcomere-length measurements were based on a grey-value profile. The volume of total mitochondria was estimated using the digitized total mitochondrial area. Specifically, the mitochondrial images obtained from the 2p channel were binarized: The grey levels higher than a selected threshold were set as “1,” and the others were set as “0.” Consequently, the number of pixels with a value of 1 was used as an estimate of the total area of the mitochondria in the image region.

### Statistical Analysis

Totally, 29 individual cells were stretched and live-imaged separately: 18 cells were in the longitudinal stretch mode; 11 cells were in the lateral stretch mode. A group of 10 cells without stretch formed the control group. To conduct statistical analysis, we considered the experimental results a binomial variate: the result with observation of sarcomeric addition (regardless of its mode, e.g., addition at the end or at the side) and the result without observation of any mode of sarcomeric addition. Accordingly, we formed two proportions: *p*_*1*_ for experiments with stretch (e.g., *p*_*1*_ = 9/11: 9 observations of sarcomeric addition in 11 independent stretch experiments) and *p*_*2*_ for experiments without stretch (e.g., *p*_*2*_ = 0/10). Because the sample size was small, we used Fisher’s exact test to examine whether the difference in the proportions between the stretched and the unstretched is statistically significant^41^. In Fisher’s exact test, the probability (P value) of getting the observed data is calculated under the null hypothesis that the proportions are the same. If the hypothesis is rejected (i.e., P < 0.01), it can be claimed that the observed sarcomeric addition is caused by the stretch. To further quantify the difference between stretched and unstretched statistically, we calculated the Agresti-Caffo 95% interval for the difference in proportions, *p*_*1*_−*p*_*2*_^42^. If the lower limit of this interval is larger than 0, the difference between stretched and unstretched conditions is statistically significant; in addition, the mid-value of the interval provides an estimate for the difference in proportions. All statistical computing was conducted under R software environment.

## Results

### 3D culture stretch model

When cell density was controlled at 7.5 × 10^5^/ml, individual neonatal CMs were sparsely distributed inside the microgrooves. Once cells started to interact with aligned collagen fibrils and physical walls, they expanded and spread into an elongated morphology along the microgroove as shown in a typical fluorescence confocal microscopic image (Fig. 1B). The height of cells inside the microgrooves was up to 14 μm (Fig. 1B). Cells outside the microgrooves (also on aligned collagen, image not shown) had a typical maximal height of 7 μm. More than 50% of the cells were observed to be not in contact with other cells; of these, a typical one that was elongated with rod-like morphology as shown in Fig. 1B was selected for SHG imaging. The SHG images presented here were reconstructed using the scans obtained during diastolic phases.

### Sarcomere-length restoration

**Figure 2:**
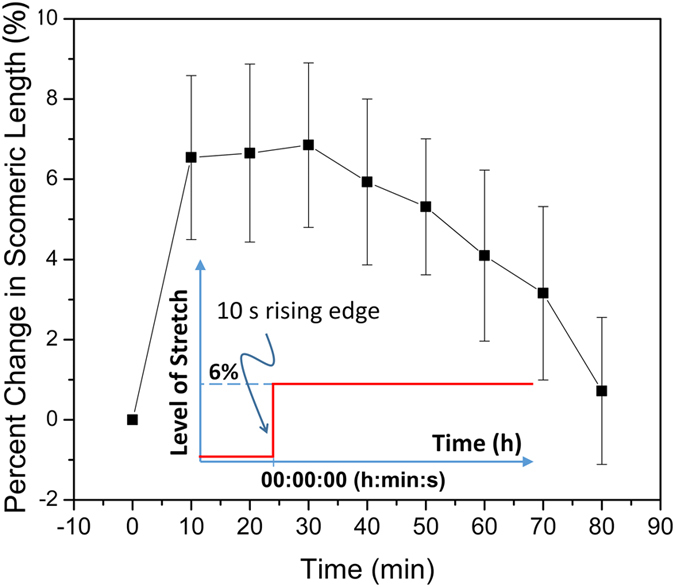
A typical curve indicating sarcomere-length restoration (n = 6). The insertion is a time-course curve of the stretch. It is a step function of time, with a very sharp rising edge ( <10 s) and a constant (static) stretching level (6%) that lasts (is sustained) during our imaging period, which was typically 4 h (acute effect).

When acute, static, sustained stretch was applied longitudinally to the PDMS substrate, the cells initially responded by elongation. Real-time phase microscopic observation of the cell-edge displacement demonstrated that the percentage a cell passively elongated was typically the same percentage as the substrate was stretched (data not presented). The time history of sarcomere-length change with stretch was studied: Length initially increased (perhaps as a result of being physically pulled by the substrate); however it then shortened towards its original value (Fig. 2). This sarcomere-length restoration varied for different cells: Maximum sarcomere-length change varied; some reached the stretch percentage (Fig. 2), and some were less than that. Variations were also seen in sarcomere-length-restoration time (i.e., between 1 and 3.5 h) and final sarcomere length. Some CMs did not reach complete restoration, as shown in Fig. 2.

### Sarcomeric addition under two stretch modes at 6%

**Figure 3:**
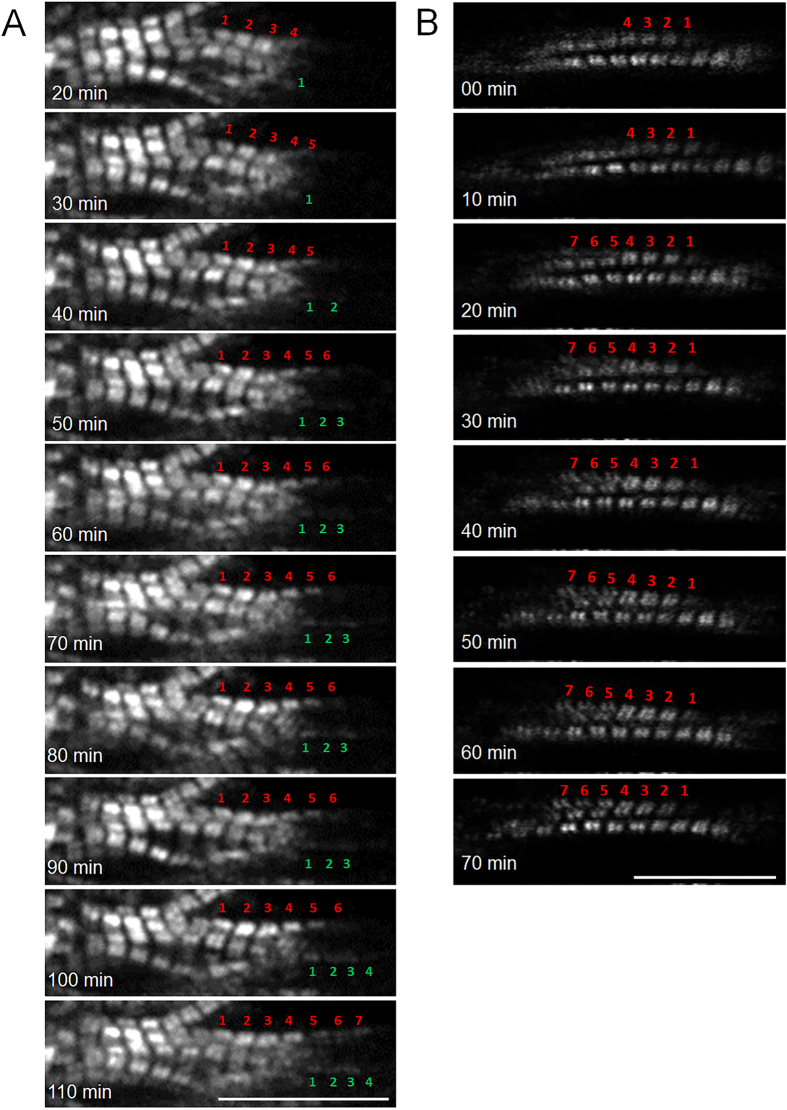
Sarcomeric addition under longitudinal stretch. (**A**) Sarcomeric addition in series at the cell end during 90 min of stretch: Six new sarcomeres were added to the ends of two myofibrils, three each. (**B**) Sarcomeric addition in parallel using an existing myofibril as a template during 0–70 min of stretch. Scale bars: 15 μm.

**Figure 4:**
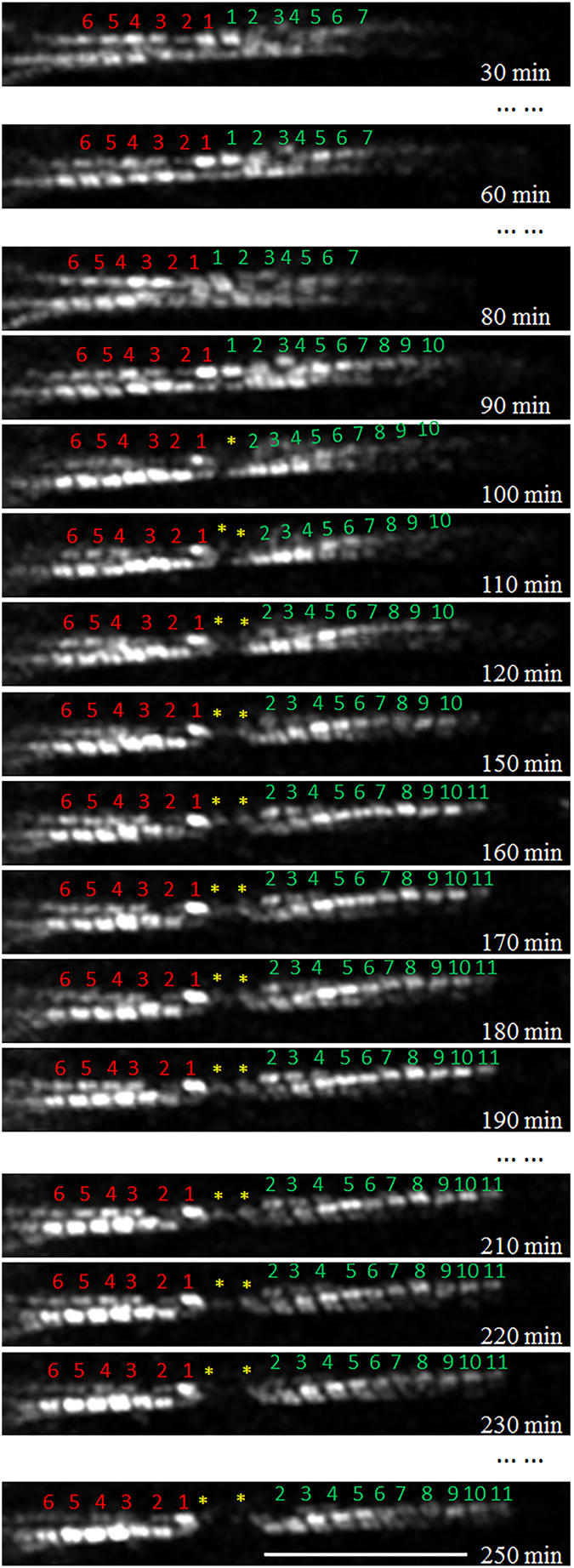
Simultaneous insertion and end addition of sarcomeres under longitudinal stretch. Approximately 30 min after the start of stretch, one myofibril increased its length through end addition of sarcomeres. During a 120-min period, four new sarcomeres were added. At approximately 100 min, the middle of the myofibril broke, and two new sarcomeres inserted during a 90-min period. At approximately 230 min, the myofibril broke again the same site. Scale bars: 20 μm.

Both longitudinal elongation and lateral extension were observed in myofibrils in the longitudinal stretch mode. Longitudinal elongation occurred when sarcomeres were sequentially added at one end of (Fig. 3A and supplemental Video 1) and/or in the middle of an existing myofibril (sarcomeric insertion, Fig. 4 and supplemental Video 2). Lateral extension occurred when sarcomeres assembled to form a new myofibril using an existing one as a template (Fig. 3B).

**Figure 5:**
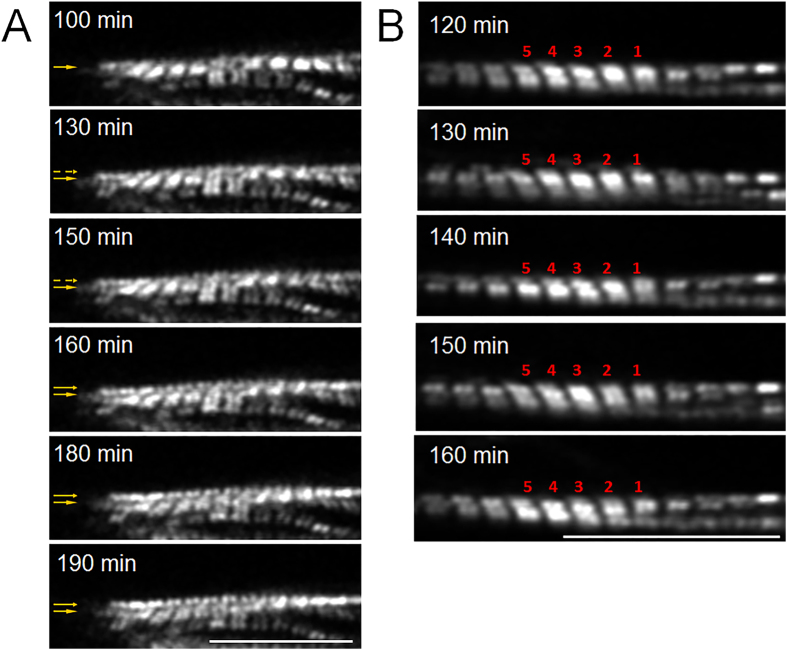
Sarcomere addition in lateral stretch mode. (**A**) A new line of sarcomeres was added with a template at 100–190 min stretch; arrows indicate the growing of new line of sarcomeres. (**B**) Sarcomere split at position 1 and 2 at 120–160 min stretch. Scale bars: 15 μm.

In the lateral stretch mode, two myofibrillar remodeling phenomena were observed: One was lateral extension through a sarcomeric assembly to form a new myofibril by using an existing one as a template, also seen with longitudinal stretch (Fig. 5A and supplemental Video 3). The other type of remodeling was myofibrillar splitting—one myofibril splitting at some point along its longitudinal axis into two (Fig. 5B and supplemental Video 4).

Confocal images of cultured CMs immunocytostained for integrin β1 to explore the possible pathway of mechanical signal transfer from the substrate to the CMs showed that integrin is distributed mainly on the plasma membrane areas that contact the microgroove surfaces (Fig. 1D); few were found on cell membranes not in contact with surfaces.

A summary of the myofibrillar remodeling of 39 single, neonatal ventricular CMs in microgrooves is presented in Table 1. Both the P value of the two-tailed Fisher’s test and the lower limit of the Agresti-Caffo 95% interval indicate that the observed sarcomeric addition correlated with stretch.

### Mitochondrial accumulation during myofibrillar remodeling

**Figure 6:**
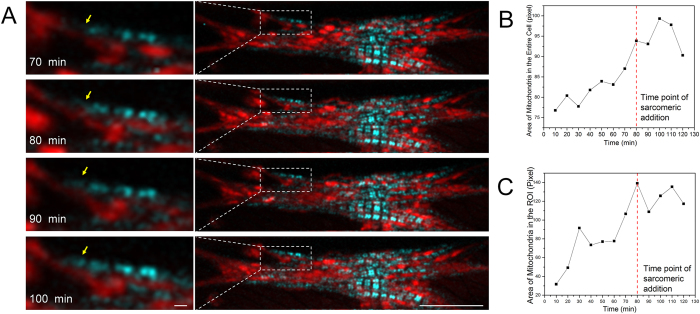
Mitochondrial clustering during sarcomeric addition measured during stretch. (**A**) Merged images of sarcomeric A-bands from the SHG channel in cyan and mitochondria from the 2 P channel in red during 70–100 min; arrows point to a newly added sarcomere; left column is a zoomed region of interest in the right column, and scale bars are 1 and 15 μm. (**B**) Change of mitochondrial area of the entire cell. (**C**) Change of mitochondrial area in ROI presented in (**A**). In both (**B**,**C**), the area was estimated through image binarization; thus the unit is number of pixels with a value of “1,” and the dashed red lines indicate the time-point of addition of a new sarcomere (80 min) during 10–120 min.

Simultaneously with the SHG channel, the 2P channel of our confocal system was used to image (use the same imaging scan as for the SHG channel) synthesis and accumulation of mitochondria (Fig. 6A), which always accompany the start of hypertrophy^43^. Near the sarcomeric addition site, dot-shape mitochondria with loose connections at the initial stage of the stretch migrated and elongated into packed parallel lines and clusters after 120 min of stretch (Fig. 6A and supplementary Video 5). The total area of mitochondria, which reflects the mitochondrial volume, increased around the sarcomeric addition site and in the entire cell body immediately after the stretch started. Sarcomeric addition occurred later than the starting point of mitochondrial accumulation (Fig. 6B,C).

## Discussion

Understanding sarcomeric addition during hypertrophy is critical to understanding and regulating cardiac cell growth under normal and overload conditions. The objective of this study was to achieve real-time observation of sarcomeric addition in CMs undergoing *in vitro*, simulated hypertrophy. Although mechanisms of hypertrophy are of considerable interest, real time observation of sarcomeric addition under hypertrophic conditions has not been previously accomplished. Because of the difficulty in isolating and then estimating the *in vivo* mechanical load and the rapid dedifferentiation process of freshly isolated adult CMs, neonatal CM culture is often used as a normal, physiological model of CM hypertrophy in which the newborn left ventricular myocytes respond to the sudden increase in systemic blood pressure at birth that is critical to postnatal survival^2^,^25^. To simulate mechanical, load-related hypertrophy, neonatal CMs are cultured on elastic membrane that is stretched during cell culture to add a specific mechanical load^23^,^44^,^45^,^46^,^47^,^48^. Based on these culture models, Russell’s group further demonstrated that neonatal CMs that form a 3D culture in the confinement of a microgroove exhibit a more *in vivo*-like response to a mechanical load than they do in a 2D culture^49^. Here, we report using similar microgrooves to confine neonatal CMs and adding an extracellular matrix component that served to produce axially aligned (along the groove) myofibrils inside each neonatal CM. This 3D alignment permitted us to observe multiple modes of sarcomeric addition in live, hypertrophying cells responding to a mechanical stimulus.

To correlate sarcomeric addition directly with changes in cell size and tension, we focused on isolated cells in culture. Our observations indicated that the geometric confinement of a groove and the aligned collagen gel that we used to coat it served to stimulate the formation of a 3D aligned myofibrillar structures (Fig. 1B). Phase and confocal microscopic observation also demonstrated that time spent in 3D culture enhanced maturation: Older neonatal CMs (after Day 4), which no longer increased in size expressed well-established and aligned cytoarchitecture with contractile myofibrils. Therefore, we conducted our stretch and sarcomeric-addition observational experiments on Day 5 in culture when actively contracting cells could be studied under sustained stretch that affected cell loading in both contractile/systolic and resting/diastolic phases.

*In vivo* studies established that volume overload produces dilation of the heart chamber and a concomitant increase in heart-wall circumference^50^; this strains each cell to its maximum level at end diastole. *In vivo* studies also showed that pressure overload causes an increase in systolic wall stress^51^; this has a very complicated distribution in the heart wall^52^ and generates a passive tensile force on each CM^53^. Although this tensile force varies in magnitude and direction with a CM’s location, it can be generally divided into two components: One along each CM’s axis stretches the cell longitudinally, and the one perpendicular to the CM’s axis stretches the cell laterally^54^. The longitudinal stretch is against active cell contraction during systole, and its role in producing hypertrophy has been studied using an isometric contraction model^55^. Here, we used sustained longitudinal and lateral stretches on the cultured neonatal cardiomyocytes 1) during the resting/diastolic phase to mimic the elevated longitudinal and lateral end diastolic strains in *in vivo* volume overload and 2) during the contractile/diastolic phase to mimic the elevated longitudinal and lateral peak passive systolic stresses. Our results using neonatal ventricular cardiomyocytes indicate that our imaging system is able to detect in real time responses to hypertrophic stimuli that previously could be only surmised from interpreting static images^56^. By live-cell imaging, we were able to confirm and extend hypotheses proposed from static images. Specifically, our data support that pressure overload does induce sarcomeric addition, but that it does so in parallel, whereas volume overload causes sarcomeric addition both in parallel (lateral surfaces of myofibrils) and in series (e.g., at the ends of myofibrils). Our data suggest that mechanisms involved include both utilization of existing sarcomeres as anchors or templates for addition and either splitting existing myofibrils for lateral expansion or inserting new sarcomeres into an existing myofibril for longitudinal expansion.

The complexity of hypertrophic processes makes it difficult or unfeasible to correlate occurrences under various levels of *in vitro* stretch to a particular type of *in vivo* hypertrophy (i.e., physiologic or pathologic). For example, in an *in vitro* hypertrophic study using a similar stretch model, Sadoshima and coworkers reported a 20.1% increase in length along the direction of the stretch (20%) and estimated that the level of stretch was still in the physiological range^26^. Conversely, our study demonstrated that for 3D-aligned neonatal ventricular cardiomyocytes, a 10% or larger stretch length caused myofibrillar breakage during our real time, SHG-confocal observations. We could not determine whether the breakage was caused only by the stretch or by the combination of stretch and laser scanning. However, in our previously conducted experiments using the same cell type and similar application of stretch, a 5% stretch could cause major hypertrophic responses at the molecular level^28^,^44^,^57^. In addition, our preliminary data showed that even a 4% longitudinal or lateral stretch performed under similar conditions could trigger dynamic addition of sarcomeres (data not shown). Consequently, in the current study, we elected to consistently use only a 6% stretch (compared to those who used much higher levels) and demonstrated that under this level of stretch, the cell would elongate accordingly (data not presented) and that the length of sarcomeres would initially increase and then go back towards their original length (Fig. 2) as reported^58^. Moreover, our real-time observational studies indicate that earliest the events of sarcomeric addition start during the process of sarcomere-length restoration. Since examination of isolated neonatal cardiomyocytes under stretch is not a generally accepted cardiac cell damage or rupture model, in our follow-on research, we will explore the potential cellular damage/rupture under a high level of cell stretch (e.g., 10%) using an end-to-end connected cell culture model, where endogenous connections and distribution of force will be examined.

Our studies revealed an *in vivo*-like hypertrophic response to change in load that was never previously seen in 2D models. For example, longitudinal myofibrillar splitting phenomena were observed only in our 3D stretching model (Fig. 5B): In cells that did not form a 3D structure, splitting was never observed. Myofibrillar splitting has long been assumed to be a mechanism for hypertrophic growth of muscle cells^15^,^59^. Signs of splitting were observed using electron microscopy in both hypertrophic cardiac^60^ and skeletal^61^ muscles. In these observations, longitudinal splitting of a myofibril was recognized when a branched myofibril exhibited partially connected Z-bands in the separation zone. Based on static images only, a splitting model was proposed for hypertrophic growth of skeletal muscle cells^62^ where myofibrils have an appearance similar to parallel cylindrical bundles, which makes myofibril splitting more easily recognized. In the heart, myofibrils (longitudinally connected sarcomeric sequences) are not long, distinctly separate filaments; rather they have a laterally connected fishnet appearance. Thus, it is more difficult to obtain convincing evidence of myofibril splitting from observation of static images. Nevertheless, a group did suggest that microtubules acted as the drivers of myofibrillar splitting in hypertrophic heart muscles^63^. Observation through our live-cell imaging system made it possible to demonstrate, in real time, the actual splitting of myofibrils in cardiomyocytes. The scanning-range limit of our instrument did not, however, let us determine whether the splitting caused by the stretch occurred at the end of a single filament or at the intersection of a branched filament.

Although the contrast of our SHG confocal does not allow us to distinguish among layers of myofibrils, its resolution (0.55 μm lateral^37^ and 1.2 μm axial^64^) does allow us to observe sarcomere-addition sites relative to the entire cell body. Our experiments show that myofibrillar splitting occurs in the central region of myofibrillar bundles, where the myofibrils, upon splitting, are less likely to bind or directly connect to the focal adhesion assemblies that mechanically link the extracellular matrix and the cell skeleton. This may be the reason that in conventional 2D culture, longitudinal myofibrillar splitting has not been observed.

It has been suggested that an external mechanical load triggers cardiomyocyte hypertrophy through outside-inside signaling mediated by integrin β1^18^,^47^. We would concur because our *in vitro* imaging data, combined with our findings on the distribution of integrin β1, indicate that 3D stretch correlates with integrin expression. Such would be consistent with the role of this receptor in transducing hypertrophic signals (mechanical or chemical) that engender longitudinal myofibrillar splitting.

In addition to myofibrillar splitting, we also observed new myofibril formation immediately beside an existing myofibril under lateral stretch. The new myofibril forms by assembling sarcomeres parallel to and in contact with an existing myofibril, a process that seems to fit the template model reviewed by Sangers^65^. However, because most of the observed template-based myofibrillogenetic processes in this study occurred in sites away from the cell membrane, there was little opportunity for growing myofibrils to use a subsarcolemmal apparatus as a template. Instead, they appeared to use an existing myofibril as a template as predicted^48^. All instances of template-based new myofibril formation that we observed, including those under longitudinal stretch, occurred in the same manner. In this template model^48^, the Z-discs of an existing myofibril served as a template upon which “Z-bodies” sequentially attached and laterally extended to form new Z-discs. This allows other components (e.g., actin and myosin filaments) to assemble longitudinally connected sarcomeres. Because the SHG imaging technique cannot visualize Z-discs, we cannot use this data to directly prove this particular template model. However, in our real time experiments, we observed three template-based processes for the lateral assembly of new myofibrils: 1) sarcomeres were sequentially added along the template myofibril; 2) sarcomeres were randomly added along the template; and 3) all sarcomeres were added at almost the same time (supplementary Video 3). Additional experiments will be required to obtain a statistically significant pattern of template-based sarcomeric addition. In addition, since SHG allows imaging of only myosin filaments, in our follow-on study, we will transfect Z-disc components (e.g., α-actinin) for live-cell imaging of the Z-disc to explore role of the Z-disc in sarcomeric addition.

Another hypertrophic response we observed in neonatal ventricular myocytes during longitudinal stretch is sarcomeric addition in series, which served to lengthen a myofibril. Two types of addition were observed. The first is sequential sarcomeric addition at the end of a myofibril in close proximity to the cell edge. Ultrastructural studies on both *in vitro* and *in vivo* hypertrophic models have demonstrated that sarcomeric addition in series is most likely to occur at the intercalated disc at the cell junctional region between two CMs in contact^66^. Because our observations were conducted on single CMs with free cell edges, our data would indicate that sarcomeric addition to the ends of existing myofibrils can occur without the cell being inserted with an intercalated disk or attached to other cells. This would suggest that neonatal myocytes, at least, have intrinsic potential to assemble myofilaments independent of cell membranes or specialized membrane structures like intercalated disks. The other type of sarcomeric addition in series under longitudinal stretch is insertion within an existing myofibril. This insertion phenomenon was previously proposed and predicted, but has been difficult to prove in static images. To insert a new sarcomere in the middle of the original myofibril, it must be disrupted or cleaved at the future insertion site. Thus, when visualized using a static image, insertion sites have been identified as broken myofibrils (possibly even artifacts). Some researchers noticed this based on static images obtained from an *in vitro* stretch assay employing high (10%) stretch or an *in vivo* tissue and proposed that myofibrillar breakage and sarcomeric synthesis may occur simultaneously within a hypertrophic cell^67^. Yu and coworkers reported static images obtained from an *in vitro* stretch assay (employing high (10%) stretch), which they interpreted as an instance of insertion of two sarcomeres into a “broken” myofibril^30^. We now show the entire insertion process in real time using a much smaller degree (6%) of stretch on the living cells. When followed dynamically, over time, the process of insertion can be clearly seen (as shown in supplemental Video 2) to be initiated by a disruption in the mid-region of the myofibril at a point where two sarcomeres would subsequently insert. For the particular insertion event shown in Fig. 4, the myofibril broke again between the two newly formed sarcomeres. Whether this represents a nucleation site for additional new sarcomeres or that the inserted sarcomeres have less integrity under load remains to be explored. Several researchers hypothesized sarcomeric insertion at Z-discs^68^,^69^ based on static observations of expanded Z-discs in hypertrophic hearts. Bishop claimed that this occurred only in overloaded hearts, not in normally developing hearts^70^. But Saetersdal and coworkers did not detect synthesis of sarcomeric proteins around the expanded Z-discs in the middle of a myofibril^67^. We also found that sarcomerogenesis appeared to occur around expanded Z-lines during *in vivo* embryogenesis in rat hearts^71^. To unequivocally prove that sarcomeric insertion occurs at Z-discs, the detailed insertion process in our cell stretch model will require further study using live-cell imaging of α-actinin in combination with SHG to simultaneously visualize Z-disc dynamics.

Mitochondria play a key role in sarcomeric addition during hypertrophy. In physiological hypertrophy, the volume of mitochondria increases to provide additional energy for the higher workload^72^. In volume overload cardiomyopathy, mitochondrial swelling is frequently observed along with increased production of reactive oxygen species, which contribute to activation of hypertrophic signaling cascades^73^,^74^. In our model, immediate increases in mitochondrial volume were detected by the 2P channel; the increase reached its maximum after sarcomeric addition started (Fig. 5B,C). Moreover, individual mitochondria formed lines in the direction of stretch, suggesting that mitochondrial clustering happens during stretch (Fig. 5A). Both results suggest a role for mitochondria in stretch-mediated hypertrophy.

## Conclusions

Major modes of sarcomeric addition were observed in real time using a custom-built SHG confocal microscope in a live cardiomyocyte culture that was stretched to mimic *in vivo* mechanical overload. These sarcomeric addition modes included 1) insertion in the middle or addition at the end of a myofibril; 2) serial addition using an existing myofibril as a template; and 3) longitudinal splitting of an existing myofibril. These modes previously could be studied based on only static microscopic images obtained from either hypertrophic hearts or cardiomyocyte cultures that mimic cell hypertrophy. The 3D cell culture system developed on a stretcher and the SHG imaging technique are unique tools for real-time analysis of cultured hypertrophic models.

## Additional Information

**How to cite this article**: Yang, H. *et al.* Dynamic Myofibrillar Remodeling in Live Cardiomyocytes under Static Stretch. *Sci. Rep.* **6**, 20674; doi: 10.1038/srep20674 (2016).

## Supplementary Material

Supplementary Information

Supplementary Video 1

Supplementary Video 2

Supplementary Video 3

Supplementary Video 4

Supplementary Video 5

## Figures and Tables

**Table 1:** Observation of sarcomeric addition in longitudinal and lateral stretch modes.

Stretch Mode	Sarcomeric Addition Mode			StatisticalAssessments
Longitudinal Stretch	Addition at the end	Addition atthe side	Insertion inthe middle	Two-tailed Fisher’s test:**P = 0.0070**Agresti-Caffo 95%interval: **(0.147, 0.686)**
Proportions	5/18	2/18	2/18	
Lateral Stretch	Myofibrillar splitting	Addition at the side		Two-tailed Fisher’s test:**P = 0.0002**Agresti-Caffo 95%interval: **(0.409, 0.963)**
Proportions	6/11	3/11		
Control	0/10			
