# Supplementary material for: Dynamic Myofibrillar Remodeling in Live Cardiomyocytes under Static Stretch
Source: Sci Rep. 2016 Feb 10;6:20674. doi: 10.1038/srep20674 (PMC4748238; doi:10.1038/srep20674)
Supplement: Supplementary Information [file srep20674-s1.doc]

**Dynamic Myofibrillar Remodeling in Live Cardiomyocytes under Static Stretch**

Huaxiao Yang^1†^, Ph.D., Lucas P. Schmidt^1†^, Zhonghai Wang^1†^, Xiaoqi Yang^1^, Thomas K. Borg^2^, Ph.D., Roger Markwald^2^, Ph.D., Raymond Runyan^2^, Ph.D., Yonghong Shao^4^*, Ph.D., Bruce Z. Gao^1^*, Ph.D.

^1^Department of Bioengineering, Clemson University, Clemson, SC, USA

^2^Department of Regenerative Medicine and Cell Biology, Medical University of South Carolina, Charleston, SC, USA

^3^Department of Cellular and Molecular Medicine, University of Arizona, Tucson, AZ, USA

^4^Key Laboratory of Optoelectronic Devices and Systems of Ministry of Education and Guangdong Province, College of Optoelectronic Engineering, Shenzhen University, Shenzhen, China

†Equal contributors as first authors

Corresponding author:

Yonghong Shao, Tel: +86 135 2884 9905; Fax: +1 86 755 2653 8556, E-mail: shaoyh@szu.edu.cn

Bruce Z. Gao, Tel: +1 864 656 0185; Fax: +1 864 656 4466, E-mail: [zgao@clemson.edu](mailto:zgao@clemson.edu)

**Legends for supplemental videos**

**Video 1** Sequential addition of new sarcomeres (shown by yellow and green arrows) under 6% longitudinal stretch at the end of existing myofibrils.

**Video 2** Sarcomeric insertion under 6% longitudinal stretch: The process is initiated by a disruption in the mid-region of the myofibril at a point where two sarcomeres (shown by two yellow arrows) subsequently inserted. Later, the myofibril broke again between the two newly formed sarcomeres.

**Video 3** Lateral extension of myofibrils, under 6% lateral stretch, through sarcomeric assembly (pointed by the yellow arrow with a small arrowhead) using an existing myofibril (pointed by the yellow arrow with a large arrowhead) as a template.

**Video 4** Myofibrillar splitting under 6% lateral stretch: One myofibril was split along its longitudinal axis into two. The slitting points are shown by yellow arrows.

**Video 5** Mitochondrial clustering (red) during sarcomeric addition (blue) under 6% longitudinal stretch. Near the sarcomeric addition site (indicated by a yellow arrow), dot-shape mitochondria with loose connections at the initial stage of the stretch migrated and elongated into packed parallel lines and clusters after 120 min of stretch.
